# ARC (NSC 188491) has identical activity to Sangivamycin (NSC 65346) including inhibition of both P-TEFb and PKC

**DOI:** 10.1186/1471-2407-9-63

**Published:** 2009-02-20

**Authors:** Luke H Stockwin, Sherry X Yu, Howard Stotler, Melinda G Hollingshead, Dianne L Newton

**Affiliations:** 1 Developmental Therapeutics Program, SAIC-Frederick Inc., NCI- Frederick, Frederick, MD 21702, USA; 2 Developmental Therapeutics Program, Division of Cancer Treatment and Diagnosis, NCI- Frederick, Frederick, Maryland 21702, USA

## Abstract

**Background:**

The nucleoside analog, ARC (NSC 188491) is a recently characterized transcriptional inhibitor that selectively kills cancer cells and has the ability to perturb angiogenesis *in vitro*. In this study, the mechanism of action of ARC was further investigated by comparing *in vitro *and *in vivo *activity with other anti-neoplastic purines.

**Methods:**

Structure-based homology searches were used to identify those compounds with similarity to ARC. Comparator compounds were then evaluated alongside ARC in the context of viability, cell cycle and apoptosis assays to establish any similarities. Following this, biological overlap was explored in detail using gene-expression analysis and kinase inhibition assays.

**Results:**

Results demonstrated that sangivamycin, an extensively characterized pro-apoptotic nucleoside isolated from *Streptomyces*, had identical activity to ARC in terms of 1) cytotoxicity assays, 2) ability to induce a G_2_/M block, 3) inhibitory effects on RNA/DNA/protein synthesis, 4) transcriptomic response to treatment, 5) inhibition of protein kinase C, 6) inhibition of positive transcription elongation factor b (P-TEFb), 7) inhibition of VEGF secretion, and 8) activity within hollow fiber assays. Extending ARC activity to PKC inhibition provides a molecular basis for ARC cancer selectivity and anti-angiogenic effects. Furthermore, functional overlap between ARC and sangivamycin suggests that development of ARC may benefit from a retrospective of previous sangivamycin clinical trials. However, ARC was found to be inactive in several xenograft models, likely a consequence of rapid serum clearance.

**Conclusion:**

Overall, these data expand on the biological properties of ARC but suggest additional studies are required before it can be considered a clinical trials candidate.

## Background

ARC (NSC 188491, SMA-491), 4-amino-6-hydrazino-7-β-d-ribofuranosyl-7H-pyrrolo-(2,3-d)-pyrimidine-5-carboxamide, is a nucleoside analog with profound *in vitro *anti-cancer activity. First identified in a high-throughput screen for inhibitors of p21 mRNA expression, subsequent experiments showed that ARC also repressed expression of hdm2 and survivin, leading to its classification as a global inhibitor of transcription [1].

As an adenosine analog, ARC is related to an important class of purine anti-neoplastics, including compounds such as fludarabine, cladribine and clofarabine, used for the treatment of chronic lymphocytic leukemia, hairy cell leukemia and refractory acute lymphoblastic leukemia, respectively [2-4]. Mechanistically, this class of drug affects quiescent and proliferating cells by impacting DNA and RNA synthesis. For example, the active metabolite of fludarabine (F-ara-ATP) competitively inhibits DNA synthesis via DNA polymerase, ribonucleotide reductase, DNA primase, and DNA ligase whilst also inhibiting RNA polymerase II [4-6]. Similarly, ARC is thought to act as an ATP competitive inhibitor of positive transcription elongation factor 2 (pTEF-b), thereby preventing phosphorylation of RNA polymerase II and blocking transcriptional elongation [1]. A recent study demonstrated that ARC inhibits replication of HIV-1 and HCV via pTEF-b, indicating it may also have utility as an anti-viral therapeutic [7].

However, several observations suggest that ARC has activities distinct from simple inhibition of transcription. For example, ARC is considerably more potent than a related pTEF-b-dependent transcriptional inhibitor, DRB (5,6-dichloro-1-β-D-ribofuranosylbenzimidazole) in inducing apoptosis and inhibiting cell viability [1,8,9]. Secondly, although ARC induces apoptosis in a wide variety of cancer cell lines in a p53-independent manner, this effect appears to be cancer selective, as transformed fibroblasts and not their 'normal' counterparts are susceptible [1,8,9]. In neuroblastoma cells, ARC was also shown to inhibit the phosphorylation of Akt at Ser-473, indicating that it may have additional kinase inhibitory activities [8,9]. Lastly, ARC was shown to inhibit *in vitro *angiogenesis assays, such as endothelial cell cord formation and motility [1]. These observations have driven the continued interest in ARC as a candidate for clinical development.

In this study, the molecular basis of ARC activity was further explored by comparison with related adenosine analogs. Results from structural homology searches identified sangivamycin and toyocamycin, two cytotoxic nucleosides isolated from *Streptomyces*, as close relatives [10]. In a panel of assays, ARC was found to have near identical activity to sangivamycin, with both compounds capable of inhibiting pTEFb, protein kinase C (PKC) and VEGF secretion. The extension of the molecular targets of ARC from pTEFb to also include PKC provides a mechanism for ARC cancer selectivity and anti-angiogenic activity *in vitro*. However, evaluation of ARC *in vivo *activity using several xenograft models yielded disappointing results, where the lack of tumor response was likely a consequence of short serum half life [11]. These data, combined with the failure of sangivamycin in clinical trials, suggest that ARC requires further development (e.g. SAR studies) before clinical trials should be considered.

## Methods

### Materials

Compounds including ARC (NSC 188491), sangivamycin (NSC 143648), toyocamycin (NSC 63701), fludarabine phosphate (NSC 312887), and 6-thioguanine (NSC 752) were obtained from the Drug Synthesis and Chemistry Branch of the Developmental Therapeutics Program, National Cancer Institute (Rockville, MD). All compounds were prepared at 40 mM in DMSO and stored at -80°C. All cell lines were from the Division of Cancer Treatment and Diagnosis (DCTD) Tumor Repository (Frederick, MD). [γ-^32^P] ATP (specific activity, 3000 Ci/mmol), [^14^C] leucine (specific activity, 306 mCi/mmol) and [5,6-^3^H] uridine (specific activity, 41 Ci/mmol) were from Perkin-Elmer (Waltham, MA), and [methyl, 1,2-^3^H] thymidine (specific activity, 128 Ci/mmol) was from GE Healthcare (Piscataway, NJ). Unless indicated in the following methods, all other chemicals were from Sigma (St. Louis, MO).

### Cytotoxicity Assays

#### Assays were conducted as follows

10^4 ^cells in 100 μL were placed into each well of a 96-well plate 24 h before treatment. Sample or buffer control (10 μL) were added to the appropriate wells and the plates were incubated at 37°C in a humidified CO_2 _incubator for the times indicated in the figure legends. Serum-containing medium was replaced with serum- and leucine-free RPMI containing 0.1 mCi of [^14^C]leucine. Incubation continued for 2 to 3 h at 37°C. The cells were harvested onto glass fiber filters using a PHD cell harvester, washed with water, dried with methanol, and counted. The results are expressed as % [^14^C]leucine incorporation into the control-treated cells. Experiments were done at least twice with triplicate determinations for each point. The IC_50 _was defined as the concentration of adaphostin required to inhibit protein synthesis by 50% relative to control-treated cells.

### RNA, DNA, and protein synthesis determination

10^4 ^cells in 100 μL were placed into each well of a 96-well plate 24 h before treatment. Sample or buffer control (10 μL) was added to the appropriate wells and the plates were incubated at 37°C in a humidified CO_2 _incubator for the times indicated in the figure legends. At the indicated times serum-containing medium was replaced with serum- and leucine-free RPMI containing either 1.3 μCi of [^3^H] uridine (RNA synthesis), 1.3 μCi of [^3^H] thymidine (DNA synthesis), or 0.03 μCi of [^14^C] leucine (protein synthesis). Incubation continued for 2 h at 37°C. The cells were harvested onto glass fiber filters using a PHD harvester, washed with water, dried with methanol, and counted. Results are expressed as % [^3^H] uridine, [^3^H] thymidine, or [^14^C] leucine incorporation into the control treated cells. Experiments were performed at least twice with triplicate determinations for each point. Where applicable, the IC_50 _was defined as the concentration of drug required to inhibit protein synthesis by 50% relative to controls.

### Apoptosis and Necrosis Determination

The percentage of apoptotic and necrotic cells in culture was determined using the Vybrant Apoptosis Assay kit (Molecular Probes, Eugene, OR) comprising an annexin VAlexa^488 ^conjugate and propidium iodide as described by the manufacturer. Acquisition and analysis of data was performed using a FACScan flow cytometer (Becton-Dickinson, Franklin Lakes, NJ) controlled by Cellquest Pro Software.

### Cell Cycle Analysis

Treated cells were harvested and washed once with PBS. The samples were resuspended in 5 mL PBS and 5 mL cold 70% ethanol were added drop wise. After 5 min incubation, the cells were centrifuged, resuspended in 10 mL cold 70% ethanol and stored at 4°C for 1 h. The cells were washed twice with 5 mL PBS and resuspended in 1 mL PBS containing 50 μg/mL propidium iodide (Molecular Probes) and 100 μg/mL RNase A (Sigma). After 1 h at 37°C, cell cycle analysis was performed using the FL3-A channel on a FACScan flow cytometer.

### Western blotting

Cell samples were washed twice with PBS and then lysed by direct addition of denaturing buffer (5 M urea, 5% SDS, 0.4 M DTT, 0.002% bromphenol blue and 0.05 M Tris HCl, pH 8.0). Samples were sonicated, centrifuged for 10 min at top speed in a microfuge, boiled for 5 min and separated using 10% NUPAGE Bis-Tris gels (Invitrogen, Carlsbad, CA) with subsequent transfer to a PVDF membrane by electroblotting. Following blocking in 2% blotto in TBST (Santa Cruz Biotechnology, Santa Cruz, CA) for 2 h, membranes were incubated overnight with either an anti phospho-(Ser) PKC substrate antibody (1:500 dilution, Cell Signaling Technology, Danvers, MA) for detection of total cell protein kinase C substrates containing phospho-serine, or with an antibody against phospho-Rpb1 CTD (Ser2/5) (1:1000 dilution, Cell Signaling Technology) for analysis of phosphorylated RNA polymerase II. Following overnight incubation, membranes were washed several times in 2% blotto in TBST, and incubated with secondary, peroxidase-conjugated antisera for 2 h. Bands were visualized using the Visualizer Western Blot Detection Reagent (Upstate, Temecula, CA) according to the manufacturer's protocol. Imaging was performed using the Kodak Image Station 2000 MM and Kodak Molecular Imaging software (Carestream Health, New Haven, CT).

### Reverse Transcription

Total RNA was isolated from cells using the RNeasy mini kit (Qiagen, Valencia, CA) and reverse transcribed using Omniscript RT (Qiagen) according to the manufacturer's instructions. A standard reaction comprised 2 μg total RNA, 0.5 mM of each DNTP, 2 μM random decamers (Ambion, Austin, TX) and 4 units of reverse transcriptase in 20 μL total volume of 1 × RT buffer. The reaction was allowed to proceed for 120 min at 37°C and the cDNA product diluted to 1 μg/mL and stored at 80°C.

### Real-time RT-PCR

SYBR Green chemistry was used to detect primer specific amplicons. For each reaction, 12.5 μL Quantitect SYBR Green PCR mastermix (Qiagen) was diluted 1:2 in DNase free water containing 5 ng cDNA and 1 μM of specific primer pair. Reactions were performed in triplicate and universal 18S RNA primers (Ambion, Austin, TX) were used to normalize cDNA amplification. The fluorochrome ROX, included in the PCR mastermix, was used as a passive reference. Reactions were performed using an ABI7500 thermocycler (Applied Biosystems, Foster City, CA). Cycling conditions consisted of a single 10 min at 95°C activation step followed by 40 cycles of 15 s at 95°C, 60 s at 60°C with fluorescence measurements taken in the elongation step of each cycle. Fold changes in expression were calculated from ΔΔCt values. For each primer pair, agarose gel electrophoresis (1%) and melting curve analysis were used to confirm the presence of a single amplicon. The generation of heatmaps from real-time PCR data was performed using the Genesis software package (Graz University of Technology, Graz, Austria). Primer sequences used in QRTPCR provided on request.

### PKC and RNA polymerase II phosphorylation

For analysis of the effect of ARC, sangivamycin, toyocamycin, fludarabine or thioguanine on endogenous or TPA-stimulated protein kinase C activity, logarithmically growing MCF7 cells were incubated with 100 μM of the drugs for 9 h. For activation of PKC, 5 μM TPA (Sigma) was included during the last 2 h of incubation. For analysis of the effects of the above drugs on RNA polymerase II phosphorylation, MCF7 cells were incubated with 100 μM of the drugs for 3 h. Due to the short exposure times, the 100 μM concentration of drug was selected although it exceeded the concentration necessary for cell growth inhibition. For both assays, lysates were prepared and immunoblotting was carried out as described above.

### Kinase assays

The activity of recombinant PKCδ (Upstate) under different conditions was determined by measuring the incorporation of ^32^P from [γ-^32^P] ATP into PKC substrate peptide 2 (Millipore, Billerica, MA) according to the manufacturer's instructions. PKA activity was determined using the PKA assay kit (Millipore), which contained purified recombinant PKA catalytic subunit and the substrate Kemptide. As a control, the PKA inhibitor peptide (Millipore) was included. The kinase activity of P-TEFb (cdk9/cyclin T1, Millipore) was determined by measuring the incorporation of ^32^P from [γ-^32^P] ATP into the synthetic PDK substrate peptide, PDKtide (Millipore), according to the manufacturer's instructions. For all of the above kinase assays, 1, 10, or 100 μM ARC, sangivamycin, toyocamycin, fludarabine or thioguanine were added to the reaction tubes for a 10 min preincubation on ice before initiation of the assay. All assays were performed at least twice and the data pooled. Results are expressed as a % activity of control treated cells.

### VEGF ELISA

The concentration of VEGF in cell-free culture supernatants was determined in triplicates using the Human VEGF Quantikine ELISA kit (R&D Systems, Minneapolis, MN) according to the manufacturer's instructions. A standard curve was generated using recombinant VEGF_165 _supplied with the kit.

### Hollow Fiber Assay

ARC efficacy was first evaluated *in vivo *using a hollow fiber animal model [12] in which polyvinylidene fluoride hollow fibers containing a panel of 12 cancer cell lines in triplicate were implanted IP and SC into mice as described previously. The agents, ARC, sangivamycin or toyocamycin, were injected IP daily for 4 days at 2 dose levels. The doses were selected based upon the single dose maximum tolerated dose (MTD). From the MTD the highest dose was calculated as the (MTD × 1.5)/4 and the low dose was 0.67 times the high dose. Twenty-four hours following the last treatment, the hollow fibers were removed and the number of viable cells determined using the stable-endpoint MTT assay as described previously [12]. A score of 2 was given for each cell line that had a ≥ 50% reduction in viable cell mass compared to the control cells. By summing the scores for all cell lines, doses, and implant sites a total score was calculated for each test agent. The maximum total score that can be achieved is 96 (12 cell lines × 2 sites × 2 doses × 2 points per positive result).

### Xenograft models

Human tumor xenograft studies were conducted to assess the antitumor activity of ARC as described previously [13]. Briefly, mice were implanted subcutaneously with MDA-MB-435 (melanoma), NCI-H522 (NSCLC), UACC-62 (melanoma), or SF-295 (CNS) human tumor cell lines. Individual tumor growth and body weights were monitored. ARC was administered by the intraperitoneal (i.p.) and intravenous (i.v.) routes using a dose volume of 0.1 ml/10 gm body weight on each of several dosing schedules. Antitumor efficacy was assessed with several endpoints including optimal % test/control (%T/C), growth delay and net log cell kill as described previously [14]. In all animal experiments, animal care procedures were in accordance with standards described in the National Institutes of Health Guide for Care and Use of Laboratory Animals.

## Results

### Analog selection

**Figure 1:**
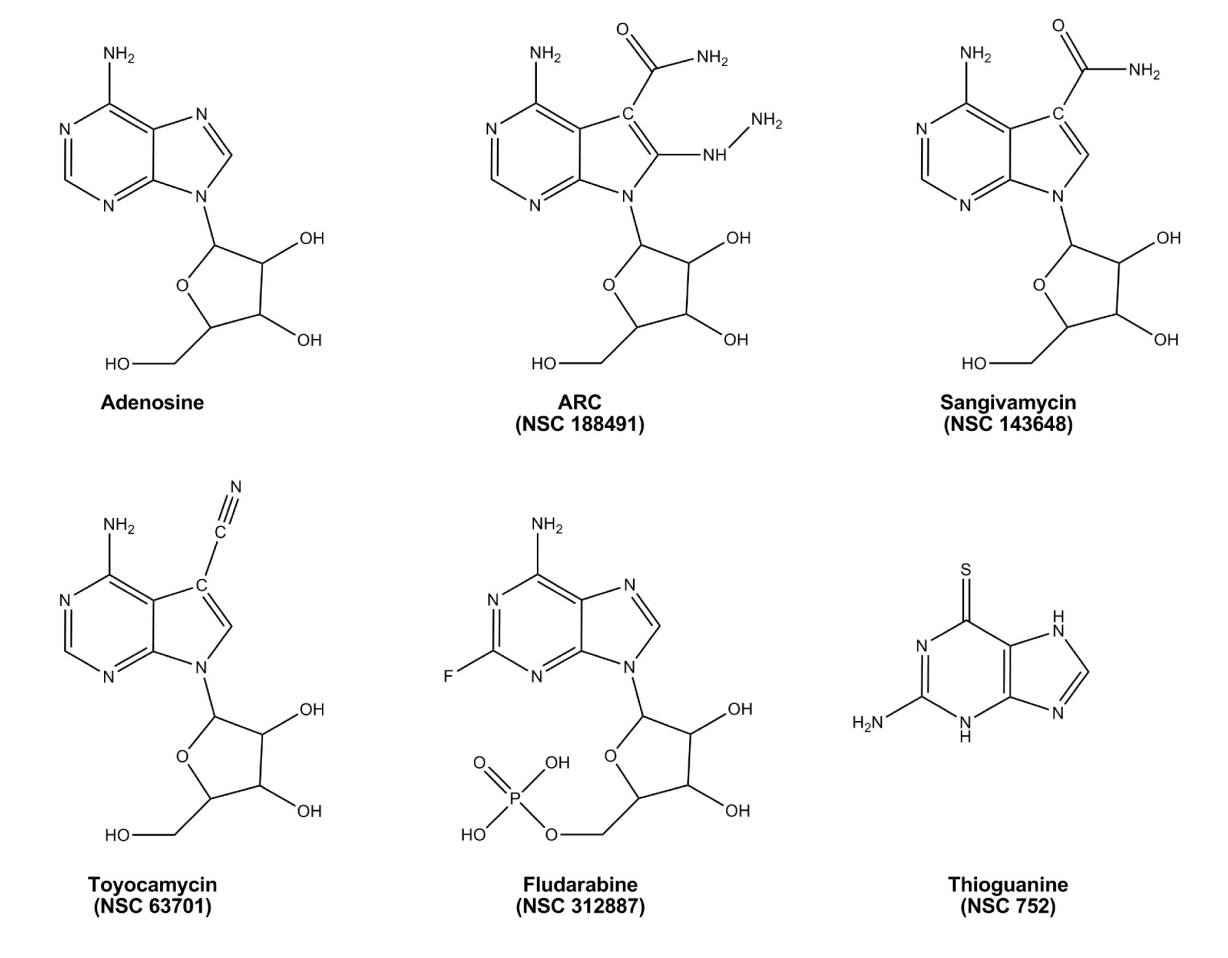
**Chemical structures of adenosine, ARC (NSC 188491), sangivamycin (NSC 143648), toyocamycin (NSC 63701), fludarabine (NSC 312887), and 6-thioguanine (NSC 752)**.

Structure-based searches of the PubChem database http://pubchem.ncbi.nlm.nih.gov were conducted to identify other nucleoside analogues with similarity to ARC. The SMILES string for ARC was obtained from the Enhanced NCI Database Browser http://129.43.27.140/ncidb2/ and searched against the PubChem database set to return any structure with >90% similarity. This search yielded sangivamycin (NSC 143648) as the primary homology hit along with several other analogs of this agent. Further reduction in stringency to 80% expanded the compound list to include several characterized anti-neoplastic adenosine analogs; including tubercidin (NSC 56408), triciribine (tricyclic nucleoside, NSC 54020) and toyocamycin (NSC 63701). Based on results showing high structural similarity, both sangivamycin and toyocamycin were selected for side-by-side biological evaluation with ARC. As controls, the structurally distinct adenosine analog fludarabine was added along with an unrelated pyrimidine anti-neoplastic 6-thioguanine. Structures for the compounds utilized in this study are shown in Fig. 1.

### Cytotoxicity, apoptosis and cell cycle analysis

**Figure 2:**
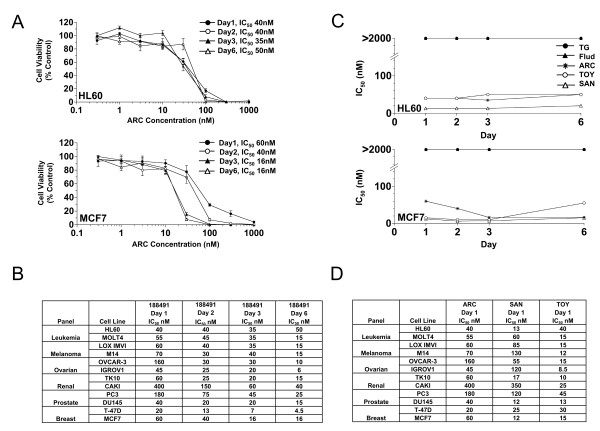
**The cytotoxic effects of ARC towards cells are similar to that of sangivamycin and toyocamycin**. A) Time course analysis of ARC on ^14^C-leucine viability of HL60 cells (upper panel) and MCF7 cells (lower panel). Data from at least 2 replicate experiments each with triplicate points were pooled and the SEM are shown when they are greater than the symbol. B) Time course analysis of ARC on a panel of 12 cell lines representative of 6 different tumor types. C) Comparison of the cytotoxic effects of ARC over 6 days with those of fludarabine (Flud), toyocamycin (TOY), sangivamycin (SAN) and thioguanine (TG) towards HL60 (upper panel) and MCF7 (lower panel) cells. The IC_50 _defined as the concentration of drug required to inhibit ^14^C-leucine viability by 50% relative to control treated cells determined from at least two pooled experiments is plotted versus time. D) Comparison of activity of ARC with sangivamycin and toyocamycin over a panel of cell lines reveals a high correlation coefficient between ARC and sangivamcin. In all panels, cells were incubated with varying concentrations (0.1 – 1000 μM) of the indicated drug.

ARC was evaluated in the context of ^14^C-leucine viability assays using two cell lines, HL60 and MCF7 (Fig. 2). In both lines, ARC was shown to have activity in the nanomolar range (Fig. 2A). The maximal effect occurred within the first 24 h of incubation for HL60 cells, while for MCF7 cells, the maximal effect did not occur until 3 days of incubation. Expanding this study to a panel of diverse tumor lines (Fig. 2B) confirmed nanomolar activity in all cell lines with a 20fold variance at day 1 (IC_50 _range 20 to 400 nM) which declined to 11fold (4.5 to 50 nM) by day 6 showing that relative cell line susceptibility gradually disappears with prolonged incubation. Following this, HL60 and MCF7 cells were treated with all 5 compounds and IC_50 _values determined over the same extended time course (1–6 days) (Fig. 2C). Results demonstrated that in both lines, the time course of cytotoxicity was similar between ARC, sangivamycin and toyocamycin, and markedly different from fludarabine and thioguanine. Having highlighted general similarities in IC_50 _for ARC, sangivamycin and toyocamycin in HL60 and MCF7 cells, the study was then extended to a panel of cell lines (Fig. 2D). Calculation of correlation coefficients showed high similarity in activity between ARC and sangivamycin (correlation coefficient, 0.87), but not between ARC and toyocamycin (correlation coefficient, 0.25). These experiments suggest for the 5 compounds, ARC and sangivamycin are the closest relatives in terms of growth inhibitory effects.

**Figure 3:**
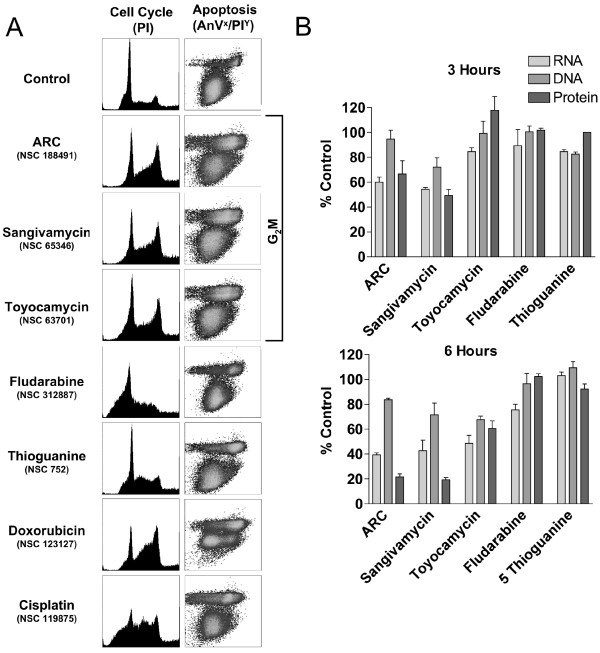
**ARC perturbs the cell cycle and induces apoptosis in a similar manner to sangivamycin and toyocamycin**. (A) HL60 cells were incubated with 2 μM of each drug for 48 h and assessed for cell cycle block by flow cytometry using propidium iodide (left panel) or for apoptosis using propidium iodide (Y axis) and Annexin V-Alexa^488 ^(X axis) (right panel). (B) ARC and sangivamycin have similar effects on RNA, DNA and protein synthesis. Cells were incubated with varying concentrations (1–10000 nM) of ARC, sangivamycin, toyocamycin, fludarabine and thioguanine for 3 to 24 h. Shown is one of three representative experiments depicting the incorporation of [^3^H] uridine into RNA, [^3^H] thymidine into DNA and [^14^C] leucine into protein after exposure of MCF7 cells to 1000 nM of the different adenosine analogs for 3 h (upper panel) or 6 h (lower panel) as a % of control treated cells.

Following this, the relative ability of ARC to induce apoptosis or perturb the cell cycle was evaluated in HL60 cells. Results from cell cycle analysis (Fig. 3A) showed marked increases in the percentage of cells in S and G_2_M phase after treatment with ARC, sangivamycin or toyocamycin, indicating progress towards a G_2_M block, whereas fludarabine demonstrated a partial block in G1 and thioguanine had little effect. Doxorubicin and cisplatin treated cells were included as controls and showed the expected S-G_2_/M and S phase blocks, respectively. In the context of apoptosis induction (Fig. 3A, right panel) ARC, sangivamycin and toyocamycin produced identical profiles with a small increase in levels of apoptosis after 48 hr treatment. Thus ARC, sangivamycin and toyocamycin behave similarly in these cell-based assays.

### RNA, DNA and Protein Synthesis

Given that adenosine analogs are recognized to have global effects on DNA and RNA synthesis, the next assays investigated the relative effects of these compounds on overall rates of DNA, RNA and protein synthesis (Fig. 3B). Results for RNA synthesis showed that, after only 3 h treatment, both ARC and sangivamycin significantly inhibited RNA synthesis (>40%), whereas the other 3 compounds had less influence (approximately 15% inhibition). Following 6 h of incubation, ARC, sangivamycin and toyocamycin inhibited RNA synthesis by approximately 55–60%, fludarabine by 25%, while thioguanine had no effect. For DNA synthesis, the effects of all compounds at both 3 h and 6 h are less profound than those observed for RNA synthesis. ARC, sangivamycin and toyocamycin inhibited DNA synthesis by 5–28% at 3 h and 16–32% at 6 h. Results from protein synthesis showed marked (35–50%) inhibition with ARC and sangivamycin at 3 h, increasing to 78–81% at 6 h. At 3 h toyocamycin, fludarabine and thioguanine had little effect on protein synthesis, whereas at 6 h toyocamycin inhibited activity by 40%. Therefore, in terms of DNA, RNA, and protein synthesis, ARC and sangivamycin have the greatest similarity, influencing RNA and protein synthesis to a greater extent than DNA synthesis.

### Inhibition of Protein Kinase C

**Figure 4:**
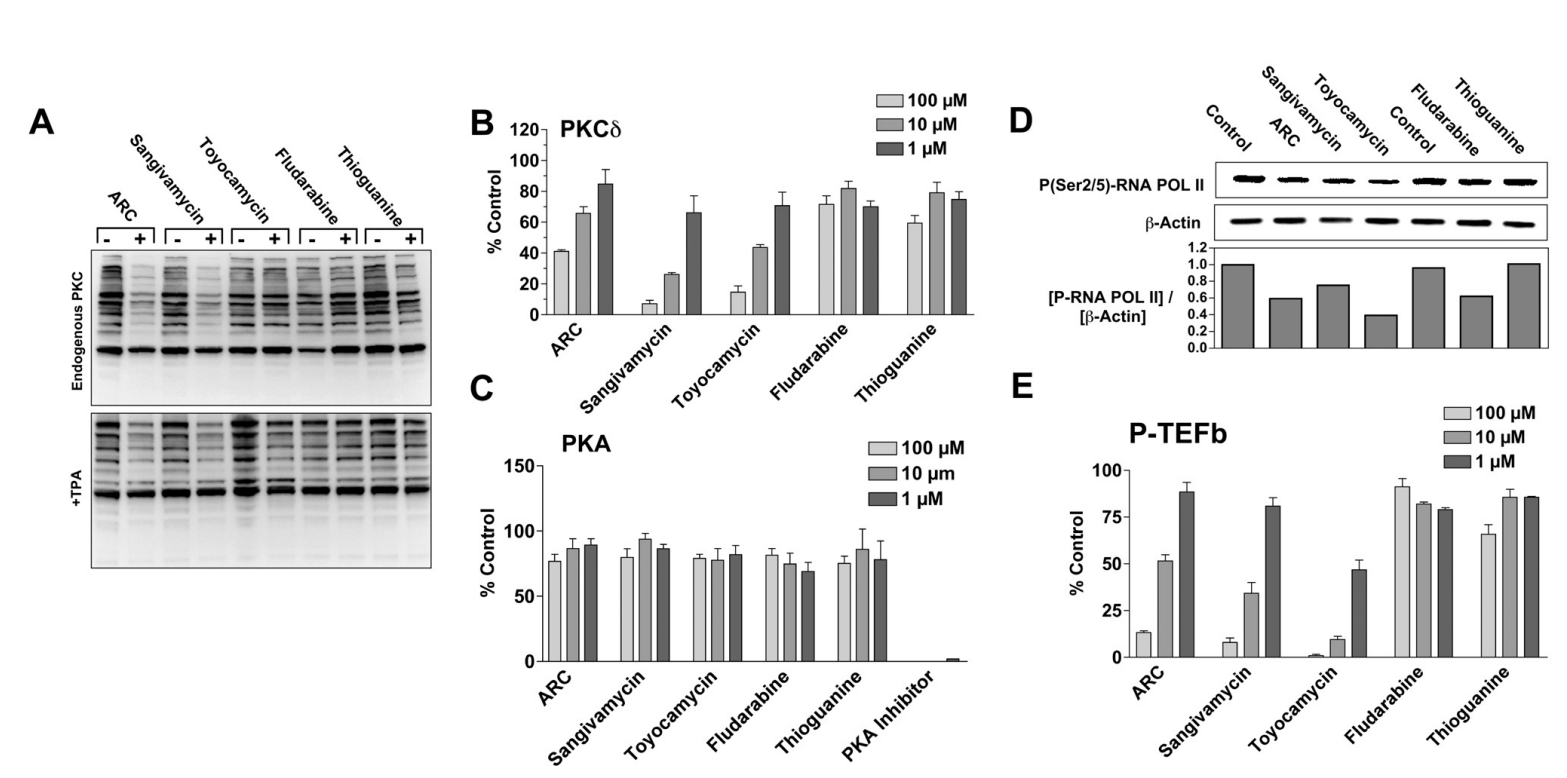
**ARC and sangivamycin inhibit PKC substrate phosphorylation, PKCδ kinase activity, RNA polymerase II phosphorylation and P-TEFb kinase activity**. A) Representative blot of the effects of ARC, sangivamycin, toyocamycin, fludarabine and thioguanine on the endogenous PKC activity (upper panel) and TPA-stimulated PKC substrate phosphorylation (lower panel). Cells were incubated for 9 h with 100 μM drug. To stimulate PKC, 5 μM TPA was included during the last 2 h of incubation. Lysates were prepared and probed for PKC substrates containing phospho-serine. B) Activity of PKCδ to incorporate ^32^P from [γ-^32^P] ATP into PKC substrate peptide 2 in the presence of the indicated concentrations of drugs. C) Activity of the recombinant PKA catalytic subunit to phosphorylate the substrate Kemptide, in the presence of the indicated concentrations of drugs and the PKA inhibitor peptide. D) Upper panel, MCF7 cells were incubated with 100 μM ARC, sangivamycin, toyocamycin, fludarabine and thioguanine for 3 h before lysates were prepared and probed for phosphorylated RNA polymerase II. Lower panel, densitometric analysis of western blots. Data is presented as the total phosphorylated RNA polymerase as a propotion of actin band intensity. E) Purified P-TEFb was incubated with substrate (PDKtide) in the absence or presence of the indicated concentrations of drug. The effects of the drugs are presented as % of control.

The parallel activities of ARC and sangivamycin in previous assays prompted further investigation into whether ARC treatment reproduces the classical activity of sangivamycin, inhibition of protein kinase C (PKC) [15]. Lysates were prepared from cells treated with 100 μM of each compound for 9 h in the presence or absence of the PKC activator, TPA (Fig. 4A). Western blots probed with an antibody which detects phosphorylated PKC substrates (anti-phospho-serine PKC substrate) illustrated that the levels of endogenous phosphorylated PKC substrate declined significantly after treatment with ARC and sangivamycin in both untreated and TPA exposed cells. Levels of phosphorylation were unaffected in cells treated with toyocamycin, fludarabine or thioguanine. We next investigated the ability of the adenosine analogs to inhibit the activity of recombinant PKCδ. ARC, sangivamycin and toyocamycin all inhibited phosphorylation of a PKC substrate peptide in the presence of [γ-^32^P] ATP (Fig. 4B). While sangivamycin is reported to be a potent inhibitor of PKC activity, it has little effect on protein kinase A. In a similar assay to that used above for PKC, none of the adenosine analogs had any effect on the ability of recombinant PKA catalytic subunit to phosphorylate its respective peptide substrate, Kemptide, whereas the control PKA inhibitor peptide completely prevented phosphorylation (Fig. 4C). Thus, both ARC and sangivamycin inhibit the kinase activity of PKC, but not of PKA.

### Inhibition of P-TEFb-mediated phosphorylation of RNA polymerase II

ARC transcriptional repression is thought to occur via inhibition of P-TEFb-mediated phosphorylation of RNA Pol II [1]. In order to determine whether sangivamycin and toyocamycin have similar properties, two assays were developed measuring either phosphorylation of endogenous RNA Pol II, or phosphorylation of the peptide, PDKtide, by recombinant P-TEFb. Results show that ARC, sangivamycin, toyocamycin and fludarabine inhibit phosphorylation of endogenous RNA Pol II (Fig. 4D) while ARC sangivamycin and toyocamycin prevent recombinant P-TEFb from phosphorylating substrate peptide (Fig. 4E). Thioguanine had no effect in either assay. These results suggest that inhibition of transcription by ARC, sangivamycin and toyocamycin is P-TEFb-dependent.

### Gene Expression Analysis

**Figure 5:**
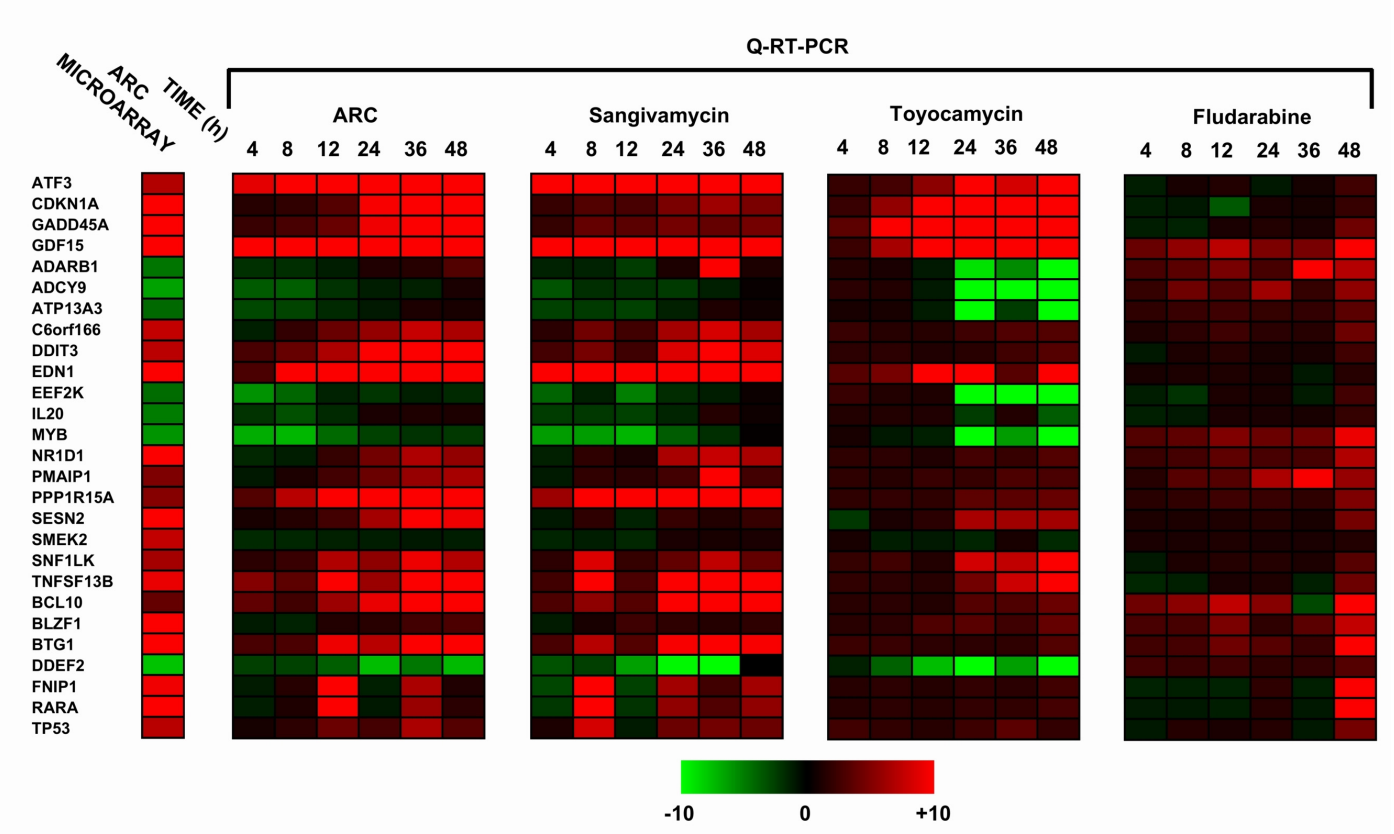
**Cells treated with ARC or sangivamycin demonstrate almost identical changes in gene expression as determined by Q-RT-PCR**. MCF7 cells were treated with the same dose (2 μM) of ARC, sangivamycin, toyocamycin or fludarabine over a time course (4, 8, 12, 24, 36 or 48 h). Total RNA was then prepared from each sample and reverse transcribed to cDNA. Each cDNA sample was then analyzed by Q-RT-PCR to determine changes in mRNA expression for 27 genes involved in processes such as the response to cellular stress, DNA damage/repair and apoptosis, that had previously been identified as modulated in microaray analysis of ARC treated MCF7 cells. Data is represented in the form of a heat map with limits of expression set at +10 fold (red) and -10 fold (green) and the primary results from microarray analysis for the same gene subset of MCF7 cells treated with ARC for 24 h shown (column, left).

Genome wide microarray analysis was performed using U133 plus 2.0 microarrays (Affymetrix) hybridized to cDNA from MCF7 cells treated with 2 μM ARC for 24 h (see Additional file 1). Genes with statistically significant ARC microarray differentials included those involved in DNA damage/stress response (example: ATF3, DDIT3, GADD45A, PPP1R15A, and SESN2), RNA metabolism (example: EEF2K, ADARB1) and cell cycle regulation (example: SNF1LK, TP53, CDKN1A). For a subset of differentially regulated transcripts, primers were designed to permit validation by real-time RT-PCR. Cells were treated with 2 μM ARC, sangivamycin, toyocamycin or fludarabine, and mRNA isolated at a range of time points (4–48 h). Real-time RT-PCR was then performed on all samples and the results presented in the form of a heat map showing fold change with treatment (Fig. 5). As shown, there is almost complete concordance between datasets for ARC and sangivamycin in terms of direction of gene regulation and the kinetics of change over time. The toyocamycin profile also has some similarity to ARC, but with what appears to be a 12–24 h lag phase. For fludarabine, the profile shows little correlation with ARC, sangivamycin or toyocamycin in terms of the direction or extent of change. These data reveal a high degree of similarity between ARC and sangivamycin in terms of the transcriptomic response to drug treatment.

### Inhibition of VEGF secretion

**Figure 6:**
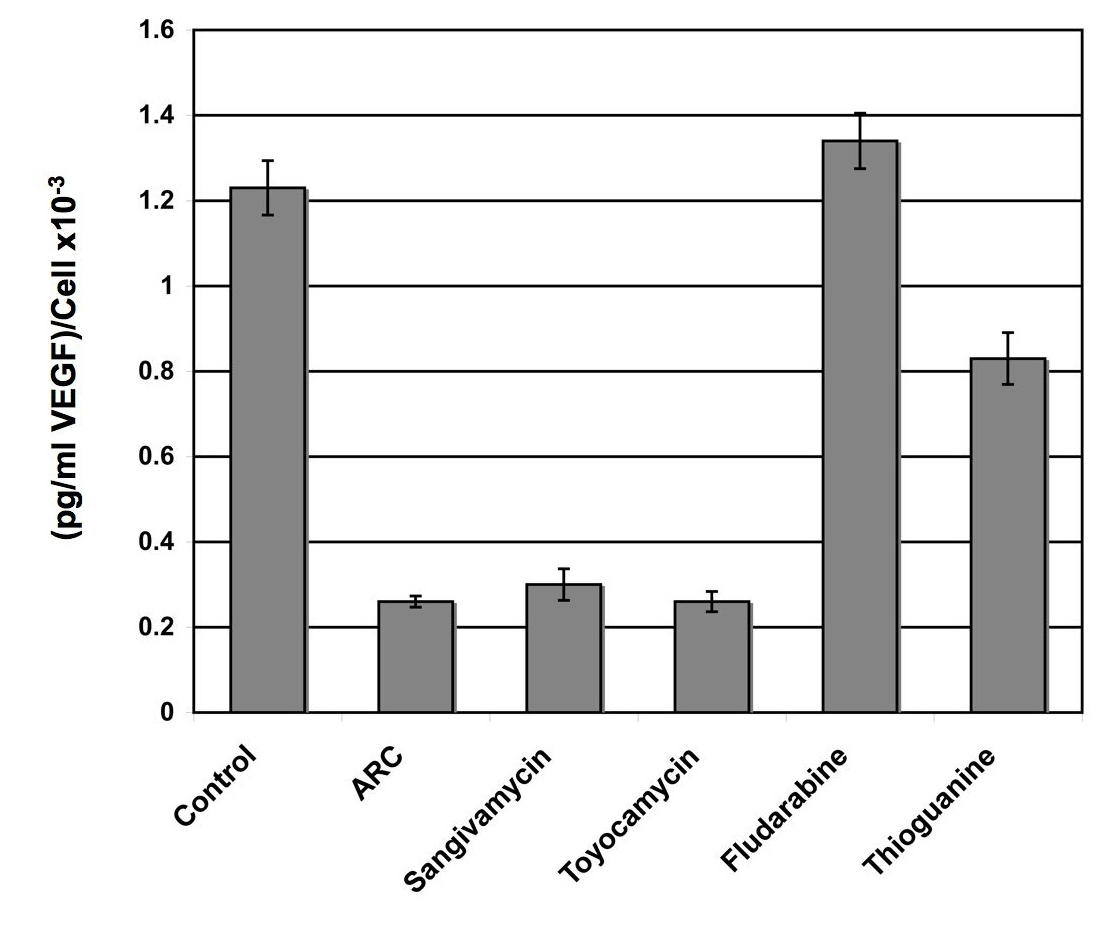
**ARC, sangivamycin and toyocamycin inhibit VEGF secretion**. MCF7 cells were grown in 6 well dishes to 50% confluence, washed twice with PBS and 2 mL fresh RPMI-1640 media added. Media was then supplemented with the appropriate drug to a final concentration of 8 μM. After 24 h supernatant was removed and any cellular debris depleted by centrifugation. Adherent cells were trypsinized and cell numbers determined. An ELISA-based method was used to measure supernatant concentrations of VEGF. To avoid changes in cell number negatively influencing levels of VEGF secretion, results are expressed as picogram VEGF/mL per cell.

ARC has been shown to possess strong antiangiogenic activity *in vitro *[1]. To compare any inherent antiangiogenic potential between drugs, an ELISA-based assay was used to quantify secretion of vascular endothelial growth factor (VEGF) from cell supernatants (Fig. 6). Values of secreted VEGF were calculated as a function of cell number to correct for the effects of cell death (pg/mL VEGF/cell). Results demonstrated a 5fold decrease in VEGF secretion for cells treated with ARC, sangivamycin or toyocamycin after 24 h. Fludarabine treatment did not appear to influence secretion whilst thioguanine decreased VEGF levels by one third. This result confirms that ARC, sangivamycin and toyocamycin all significantly inhibit secretion of this important angiogenic factor.

### *In vivo *Activity

ARC was next evaluated *in vivo *using a hollow fiber assay and xenograft models. The *in vivo *doses evaluated for the ARC, sangivamycin and toyocamycin reflect differences in the inherent mouse toxicity of each agent (see methods for dose selection). Interestingly, as reflected in the administered doses, ARC is almost 50% less toxic acutely to mice than sangivamycin. In hollow fiber assays, ARC at 75 and 50 mg/kg/injection, had a total score of 54 (IP score 44; SC score 10) compared to a total score of 50 for sangivamycin (9 and 6 mg/kg/injection; IP score 42; SC score 8) and 28 for toyocamycin (0.9 and 0.6 mg/kg/injection; IP score 26; SC score 2). Total scores of 20 or greater are considered indicative of potential for evaluation in xenograft models. Thus, in the hollow fiber assay, ARC and sangivamycin have similar activity and both are approximately twice as active as toyocamycin.

ARC efficacy was then investigated in four xenograft animal models; 1) H522 non-small cell lung cancer, 2) MDA-MB-435 melanoma, 3) SF-295 CNS, and 4) UACC-62 melanoma. Treatment was administered i.p. at doses ranging from 8–100 mg/kg/dose given once or twice per day for 5–10 days or once every other day for a total of 5 doses. Treatment was administered i.v. at doses ranging from 32–72 mg/kg/dose given once or twice per day for 5–10 days or once every other day for a total of 5 doses. Toxicity was noted in the groups receiving 100 or 67 mg/kg/dose i.p. twice daily for 5 days. ARC did not demonstrate efficacy against any of the 4 xenograft tumors in 5 separate experiments at any dose, route or schedule evaluated. Efficacy was defined as a %T/C value of 40% or less at any tumor measurement time point [14].

## Discussion

ARC (NSC 188491) undoubtedly has profound anti-cancer activity *in vitro*. However, the novelty of this agent when compared to other adenosine anti-metabolites remains unclear. Structure-based searches revealed considerable similarity with sangivamycin and toyocamycin, two extensively studied pyrrolopyrimidine antibiotics with anti-cancer activity [10,16,17]. Analysis of these compounds in viability assays against a panel of cell lines showed a high correlation in cytotoxicity between ARC and sangivamycin. Both compounds also blocked the cell cycle in G_2_/M and had similar inhibitory effects on DNA, RNA and protein synthesis. For toyocamycin, the correlation with ARC cytotoxicity was less robust and the pattern of inhibition of RNA, DNA and protein synthesis differed from that of ARC and sangivamycin. The activity differential between sangivamycin and toyocamycin has been described previously [18]. This report inferred that differentials were a consequence of toyocamycin primarily inhibiting rRNA processing whereas sangivamycin inhibited protein synthesis. Still, the degree of concordance between ARC and sangivamycin was surprising. Analysis of transcriptomic changes occurring after treatment with ARC, sangivamycin or toyocamycin added significance to the correlation between ARC and sangivamycin given that these two compounds perturb identical groups of genes and the slope of change over a time course was almost superimposable between the two drugs. For toyocamycin, the presence of a 1224 h lag period in mRNA response again hints towards functional differences with ARC.

Given results suggesting a correlation between ARC and sangivamycin, we then speculated as to whether overlap existed for the molecular targets inhibited by each agent. The seminal activity of sangivamycin concerns inhibition of protein kinase C, whereas ARC had been shown to repress RNA polymerase II activity via P-TEFb [1,19]. In this regard, a central finding was that ARC is also capable of inhibiting PKC. This fact provides an alternate molecular target responsible for ARC anti-cancer activity. PKC isoenzymes have been suggested as attractive therapeutic targets for anticancer therapies [20]. Activation of PKC has been shown to positively affect tumor cell proliferation, invasion, metastasis, and tumor angiogenesis [21]. If we assume that PKC is a molecular target for ARC activity then this may explain the selective ability of ARC to kill cancer cells, since other PKC inhibitors have also been shown to have greater activity against cancer cells than their 'normal' counterparts [22].

Interestingly, in a recent study sangivamycin was shown to exert differential anti-tumor effects in drug-sensitive MCF7/wild type (WT) cells and MCF7/Adriamycin (MCF7/ADR) resistant cells [23]. Wild-type MCF7 cells were observed to check point in G_2_/M with sangivamycin without significant apoptosis, mirroring the HL60 results obtained here. The cell cycle arrest can be explained in part by the increase in p53 tumor suppressor protein thought to be essential for maintaining a G_2 _arrest [24]. ARC has also been shown to cause an accumulation of p53, see [1] and Fig 5. For the MCF7/ADR cells sangivamycin also induced an early cell cycle arrest at the G_2_M phase but this was followed by massive apoptotic cell death most likely due in part to expression of inactive p53 protein.

Additionally, both ARC and sangivamycin have anti-angiogenic activity. Sangivamycin has been shown to inhibit 1) growth of human umbilical vein endothelial cells (HUVEC) *in vitro*, 2) cord formation of HUVEC on matrigel, 3) *in vivo *formation of new blood vessels in the chicken chorioallantoic membrane assay and 4) tumor induced angiogenesis within the mouse dorsal skin [25,26]. Similarly, ARC inhibits the growth of HUVEC, cord formation of HUVEC on matrigel and reduces the motility of HUVEC toward the chemoattractant, VEGF [1]. As shown here, both ARC and sangivamycin inhibit VEGF secretion. Recently, it has been shown that PKC promotes the angiogenic activity of HUVEC by enhancing VEGF expression [27]. PKC inhibitors were shown to inhibit PMA enhanced cord formation of HUVEC on matrigel and PMA induced expression of VEGF. Therefore, inhibition of PKC provides a possible molecular basis for the anti-angiogenic activity attributed to ARC [1].

The identical behavior of ARC and sangivamycin is interesting given that the latter has been extensively studied in clinical trials. Several reports exist of Phase I trials of sangivamycin in patients with a range of malignancies [28-30]. Likewise, archives of the Developmental Therapeutic program (DTP) of the NCI contain details of additional attempts at sangivamycin clinical development (unpublished data). The Eastern Cooperative Oncology Group treated 47 patients with a total dose ranging from 0.1–2.8 mg sangivamycin/kg and observed no objective or subjective responses, where the major side effect was hypotension. The Veterans Administration Chemotherapy Group treated 15 patients; no therapeutic or toxic side effects were observed in the first 10 patients treated with 50 mg/kg/D × 7. The next 2 patients received 100 mg/kg/D × 6 or 10 (total dose of 36 or 60 mg). The higher dose proved fatal. The last dosage regimen was 150 mg/kg/D × 7, which proved fatal to the final 3 patients with no tumor responses observed. Lastly, the New England Medical Center treated 4 cases of pediatric leukemia. Three of the four children suffered cardiotoxicity. This led to a review of the Phase I records in which it was discovered that 2/16 adult patients showed development of A-V conduction defects. Clinical studies were then halted pending cardiac toxicity studies in animals. In the feline and canine acute studies, EKG showed no evidence of cardiotoxicity. Subacute studies using immature canines however, revealed EKG changes following the second course of sangivamycin administration. Interestingly, tentative support for potential ARC cardiac toxicity came from Ingenuity Pathways IPA-Tox analysis http://www.Ingenuity.com of the differentially expressed transcript list from ARC treated cells (results not shown). Here, the spreadsheet of transcripts was imported into ingenuity allowing the software to automatically determine whether changes in expression of input genes are associated with a defined toxicity (for further details see – http://www.ingenuity.com/library/videos/tox_video/Toxicology_video.htm). Results showed that cardiac necrosis/cell death was the primary predicted toxicology for the input list, indicating that ARC and sangivamycin may have overlapping adverse effects.

As regards *in vivo *efficacy, ARC was active in hollow fiber assays but not in several xenograft models. In the hollow fiber assay, activity was primarily restricted to tumor cells growing within the peritoneal cavity (i.p. score of 42 vs s.c. score 10). This is the same physiologic site into which the test article was injected. Thus, tumor cells were exposed to high concentrations of ARC immediately post-injection and systemic bioavailability was not an issue in this assay. In contrast, administration of ARC (i.p or i.v) to animals bearing human tumor xenografts requires systemic distribution for antitumor activity to be detected, thus drug metabolism plays an important role in determining activity, providing a possible explanation for the failure of ARC in xenograft studies. Evidence to support this concept comes from a recent study showing that ARC is relatively unstable in serum [11]. Therefore, In light of this pharmacologic data, it appears likely the absence of antitumor activity resulted from inadequate tumor exposure as a conseqeunce of ARC metabolism.

## Conclusion

In conclusion, this study establishes that ARC has identical *in vitro *biological activity to the classical PKC inhibitor sangivamycin. Furthermore, in defining ARC as an inhibitor of both PKC and RNA polymerase II mediated transcription through P-TEFb, we provide a molecular basis for *in vitro *cancer selectivity and anti-angiogenic activity. Importantly, although ARC retains some activity in hollow fiber assays, the compound was inactive in xenografts. Pharmacokinetic studies hint that the lack of serum stability may be responsible for lack of *in vivo *activity. It is also noteworthy that repeated clinical trials of sangivamycin failed to demonstrate efficacy. Therefore, although *in vitro *activity is compelling, additional studies are required before ARC can be considered for clinical development.

## Competing interests

The authors declare that they have no competing interests.

## Authors' contributions

LHS performed cell cycle analysis, apoptosis assays, generated the microarray data. LHS and DLN produced the manuscript. SXY provided technical/assay support. MGH and HS provided *in vivo *data and DLN was responsible for cell viability, DNA/RNA/Protein synthesis assays and kinase inhibition assays. All authors read and approved the final manuscript.

## Pre-publication history

The pre-publication history for this paper can be accessed here:

http://www.biomedcentral.com/1471-2407/9/63/prepub

## Supplementary Material

Additional file 1 **Results from Microarray Analysis.** The Genesifter suite (VizX labs, Seattle, WA) was used for analysis of microarray data. In brief, compressed .CEL files containing array data were loaded into the Genesifter web portal http://www.genesifter.net. Using the advanced upload function, probe-level data was then compiled, normalized and transformed using GC-RMA. Pairwise analysis was conducted using duplicate control MCF7 arrays versus duplicate arrays from ARC treated (2 μM, 24 hours) MCF-7 cells. The following criteria were applied to filter the differentially expressed transcript list, a fold change of >4 and a Wilcoxon rank sum test where p < 0.05 with Benjamini-Hochberg estimation of false discovery rate (FDR). The list of differentially regulated transcripts was then exported into excel for gene ontogeny analysis. Raw data files for microarray analysis (.CHP and .CEL) can be downloaded from http://www.ncbi.nlm.nih.gov/projects/geo/query/acc.cgi?acc=GSE13477. Click here for file

## References

[B1] Radhakrishnan SK Gartel AL A novel transcriptional inhibitor induces apoptosis in tumor cells and exhibits antiangiogenic activity Cancer Res 2006 66 6 3264 3270 10.1158/0008-5472.CAN-05-3940 16540679

[B2] Rai KR Peterson BL Appelbaum FR Kolitz J Elias L Shepherd L Hines J Threatte GA Larson RA Cheson BD Schiffer CA Fludarabine compared with chlorambucil as primary therapy for chronic lymphocytic leukemia N Engl J Med 2000 343 24 1750 1757 10.1056/NEJM200012143432402 11114313

[B3] Piro LD Carrera CJ Carson DA Beutler E Lasting remissions in hairy-cell leukemia induced by a single infusion of 2-chlorodeoxyadenosine N Engl J Med 1990 322 16 1117 1121 1969613 10.1056/NEJM199004193221605

[B4] Robak T Lech-Maranda E Korycka A Robak E Purine nucleoside analogs as immunosuppressive and antineoplastic agents: mechanism of action and clinical activity Curr Med Chem 2006 13 26 3165 3189 17168705 10.2174/092986706778742918

[B5] Gandhi V Plunkett W Cellular and clinical pharmacology of fludarabine Clin Pharmacokinet 2002 41 2 93 103 10.2165/00003088-200241020-00002 11888330

[B6] Huang P Plunkett W Action of 9-beta-D-arabinofuranosyl-2-fluoroadenine on RNA metabolism Mol Pharmacol 1991 39 4 449 455 1708088

[B7] Nekhai S Bhat UG Ammosova T Radhakrishnan SK Jerebtsova M Niu X Foster A Layden TJ Gartel AL A novel anticancer agent ARC antagonizes HIV-1 and HCV Oncogene 2007 26 26 3899 3903 10.1038/sj.onc.1210158 17173067

[B8] Radhakrishnan SK Halasi M Bhat UG Kurmasheva RT Houghton PJ Gartel AL Proapoptotic compound ARC targets Akt and N-myc in neuroblastoma cells Oncogene 2007 27 5 694 699 17724478 10.1038/sj.onc.1210692

[B9] Bhat UG Gartel AL Differential sensitivity of human colon cancer cell lines to the nucleoside analogs ARC and DRB Int J Cancer 2007 122 6 1426 1429 17999411 10.1002/ijc.23239

[B10] Rao KV Structure of sangivamycin J Med Chem 1968 11 5 939 941 10.1021/jm00311a005 5748694

[B11] Mao Li WW Rayburn E Wang H Hill D Covey J Zhang R Preclinical pharmacological evaluations of SMA-491, a nucleoside analogue related to sangivamycin AACR Meeting Abstracts 2008 2008 3306

[B12] Hollingshead MG Alley MC Camalier RF Abbott BJ Mayo JG Malspeis L Grever MR In vivo cultivation of tumor cells in hollow fibers Life Sci 1995 57 2 131 141 10.1016/0024-3205(95)00254-4 7603295

[B13] Alley MC Hollingshead MG Dykes DJ Maud WR Teicher BA, Andrews PA Human tumor xenograft models in NCI drug development Anticancer drug development guide: preclinical screening, clinical trials, and approval 2004 2 Totowa, N.J.: Humana Press 125 152

[B14] Plowman J Harrison SD Dykes DJ Jr Paull KD Narayanan VL Tobol HK Martin J Griswold DP Jr Preclinical antitumor activity of an alpha-picoline derivative, penclomedine (NSC 338720), on human and murine tumors Cancer Res 1989 49 8 1909 1915 2702634

[B15] Osada H Sonoda T Tsunoda K Isono K A new biological role of sangivamycin; inhibition of protein kinases J Antibiot (Tokyo) 1989 42 1 102 106 2921215 10.7164/antibiotics.42.102

[B16] Nishimura H Katagiri K Sato K Mayama M Shimaoka N Toyocamycin, a new anti-candida antibiotics J Antibiot (Tokyo) 1956 9 2 60 62 13345725

[B17] Tolman RL Robins RK Townsend LB Pyrrolo[2,3-d]pyrimidine nucleoside antibiotics. Total synthesis and structure of toyocamycin, unamycin B, vengicide, antibiotic E-212, and Sangivamycin (BA-90912) J Am Chem Soc 1968 90 2 524 526 10.1021/ja01004a076 5634627

[B18] Cohen MB Glazer RI Comparison of the cellular and RNA-dependent effects of sangivamycin and toyocamycin in human colon carcinoma cells Mol Pharmacol 1985 27 3 349 355 2579317

[B19] Loomis CR Bell RM Sangivamycin, a nucleoside analogue, is a potent inhibitor of protein kinase C J Biol Chem 1988 263 4 1682 1692 3338987

[B20] Teicher BA Protein kinase C as a therapeutic target Clin Cancer Res 2006 12 18 5336 5345 10.1158/1078-0432.CCR-06-0945 17000666

[B21] Podar K Raab MS Chauhan D Anderson KC The therapeutic role of targeting protein kinase C in solid and hematologic malignancies Expert Opin Investig Drugs 2007 16 10 1693 1707 10.1517/13543784.16.10.1693 17922632

[B22] Gonindard C Bergonzi C Denier C Sergheraert C Klaebe A Chavant L Hollande E Synthetic hispidin, a PKC inhibitor, is more cytotoxic toward cancer cells than normal cells in vitro Cell Biol Toxicol 1997 13 3 141 153 10.1023/A:1007321227010 9088624

[B23] Lee SA Jung M The nucleoside analog sangivamycin induces apoptotic cell death in breast carcinoma MCF7/adriamycin-resistant cells via protein kinase Cdelta and JNK activation J Biol Chem 2007 282 20 15271 15283 10.1074/jbc.M701362200 17371872

[B24] Bunz F Dutriaux A Lengauer C Waldman T Zhou S Brown JP Sedivy JM Kinzler KW Vogelstein B Requirement for p53 and p21 to sustain G2 arrest after DNA damage Science 1998 282 5393 1497 1501 10.1126/science.282.5393.1497 9822382

[B25] Ohno O Shima Y Ikeda Y Kondo SI Kato K Toi M Umezawa K Selective growth inhibition by sangivamycin of human umbilical vein endothelial cells Int J Oncol 2001 18 5 1009 1015 11295049 10.3892/ijo.18.5.1009

[B26] Komi Y Ohno O Suzuki Y Shimamura M Shimokado K Umezawa K Kojima S Inhibition of tumor angiogenesis by targeting endothelial surface ATP synthase with sangivamycin Jpn J Clin Oncol 2007 37 11 867 873 10.1093/jjco/hym115 17956898

[B27] Xu H Czerwinski P Hortmann M Sohn HY Forstermann U Li H Protein kinase C {alpha} promotes angiogenic activity of human endothelial cells via induction of vascular endothelial growth factor Cardiovasc Res 2007 78 2 349 355 18056764 10.1093/cvr/cvm085

[B28] Cavins JA Hall TC Olson KB Khung CL Horton J Colsky J Shadduck RK Initial toxicity study of sangivamycin (NSC-65346) Cancer Chemother Rep 1967 51 4 197 200 4318252

[B29] Slavik M Nucleoside analogs in the treatment of neoplastic and nonneoplastic diseases Ann N Y Acad Sci 1975 255 266 268 10.1111/j.1749-6632.1975.tb29234.x 1059360

[B30] Robins RK Revankar GR Purine analogs and related nucleosides and nucleotides as antitumor agents Med Res Rev 1985 5 3 273 296 10.1002/med.2610050302 3894832

